# Thermomechanics and Thermophysics of Optical Fiber Polymer Coating

**DOI:** 10.3390/polym18020271

**Published:** 2026-01-20

**Authors:** Aleksandr N. Trufanov, Anna A. Kamenskikh, Yulia I. Lesnikova

**Affiliations:** 1 Department of Computational Mathematics, Mechanics and Biomechanics, Perm National Research Polytechnic University, 614990 Perm, Russia; ant@pstu.ru (A.N.T.); ulesig@gmail.com (Y.I.L.); 2 Laboratory of Digital Engineering of Mechanical Engineering Processes and Production, Perm National Research Polytechnic University, 614990 Perm, Russia

**Keywords:** polarization-maintaining fiber, Panda fiber, polymer, protective coating, geometric configuration, material behavior models, deformation behavior, thermomechanics, viscoelasticity

## Abstract

The viscoelastic properties of ultraviolet radiation-curable polymer coatings of optical fibers were studied experimentally and numerically. The test setup was completed, and a series of natural experiments were conducted for an extended temperature range from −110 °C to +120 °C using a dynamic mechanical analyzer (DMA). Discrete dependencies of the complex modulus on temperature and frequency of kinematic loading were obtained. The problem of multiparametric optimization was solved. Defining relations were obtained for protective coating polymers, making it possible to describe the thermomechanical behavior of the glass-forming materials under consideration in a wide temperature range, including relaxation transition. The optimal solution was found for 18 series terms at the selected reference temperature *T*_r_ = −70 °C, *C*_1_ = 20.036, and *C*_2_ = 32.666 for the DeSolite 3471-1-152A material. The optimal solution was found for 60 series terms at the selected reference temperature *T*_r_ = 0 °C, *C*_1_ = 40,242.2827, and *C*_2_ = 267,448.888 for the DeSolite DS-2015 material. The models were verified according to the data of creep experiments. The capabilities of the viscoelastic model were demonstrated by the example of a numerical experiment on free thermal heating/cooling of a Panda-type optical fiber.

## 1. Introduction

From 1996 to the present day, there has been active development in the field of microstructural and special optical fibers [1]. The polarization-maintaining fibers (PMFs) can be distinguished as a separate class of optical fibers. They are used as sensing elements in sensor systems [2]. A special single-mode optical fiber of the Panda-type refers to a PMF. Panda-type optical fibers serve as a sensing element in many sensors [3,4,5], including optical gyroscopes [6].

Special optical fibers consist of doped fused silica protected by 1–2 polymer coating layers [7,8]. Optical fibers are drawn from preforms at high temperatures [9,10]. The drawing process can create surface microdefects, which significantly reduce fiber strength [11]. Polymer coatings are applied to minimize defects and protect fibers from mechanical damage, microbending, and harsh environments [9,12]. But polymer protective coatings can negatively affect the optical characteristics of fibers due to the stability of polymer properties only in a limited temperature range [13]. The prolonged thermal cycling can lead to reduced resistance to microbending, thermal shrinkage, the formation of axial stresses in the fiber, and degradation or aging of polymers.

First, single-layer acrylate protective coatings were developed [12]. Later, two-layer coatings became the standard. In these, the outer layer of the coating provides protection from the mechanical impacts of the internal damping layer and the fiber itself [13]. The coating thickness ranges from tens to hundreds of microns. The thickness, the layer ratio, and the material properties all affect fiber performance [14]. Primary coatings must have a low glass transition temperature and a low elastic modulus at subzero temperatures [15]. Secondary coatings must be stronger and have a higher glass transition temperature to protect the inner layers over the entire operating temperature range. The composition of the polymer coating [16] and the technology of its curing on the fiber [17] also have a significant effect on the thermomechanical behavior of the materials. This affects the optical characteristics of the fiber and the sensors. The mechanical strength and stability of the fiber (ability of the fiber to maintain high operational reliability) depend on the properties of the protective coating and the phenomena observed at the interfaces of the glass base and coatings of different types [18].

Studying the thermomechanical properties of coatings is relevant for material selection according to operating conditions and performance characteristics [10,12], rationalizing their application technology [19], and creating numerical models, digital shadows, and digital twins of fibers and sensor systems [6,20,21]. Creation of effective digital models of optical systems implies not only a large amount of empirical studies, but also the formation of behavioral models of the materials over a wide range of temperatures and strain rates. Most researchers are considering fiberoptic structures, devices, and sensors in thermoelastic formulations [22,23,24,25]. Simplified cross-sectional geometries, including those without protective coatings, are considered [24,25,26]. But polymer protective coatings have a significant impact on the performance and functionality of the optical fibers [27]. It is known that relaxation processes, for instance, softening of polymers at high temperatures and glass transition at low temperatures, affect the behavior of the materials and systems in which they are used [28]. The thermoelastic formulation does not allow for the correct description of various effects that occur under conditions of thermal force, vibration, or other complex types of loading.

The key goal of the current study is to propose a methodology for constructing viscoelastic models and demonstrate their ability to describe complex nonlinear effects occurring in polymers. The models are based on experimental studies for two widely used polymer coating materials for optical fibers. The constructed models expand the capabilities of numerical studies of optical fibers and sensors. The methodology for constructing viscoelastic models can be applied to other polymer materials used to create film polymer coatings.

## 2. Materials and Methods

### 2.1. Panda-Type Optical Fiber

**Figure 1:**
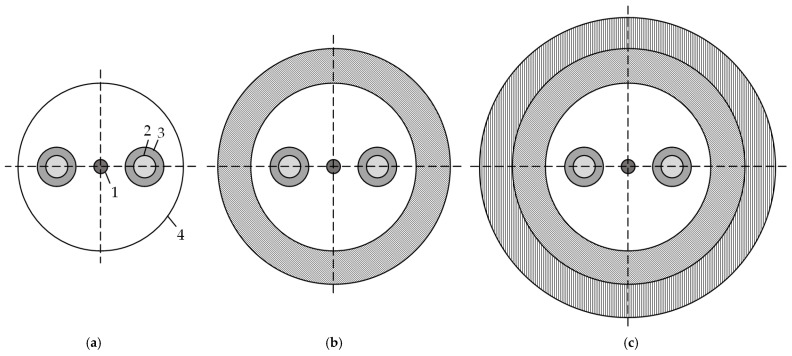
Panda-type optical fiber: (**a**) is a fiber without a protective coating; (**b**) is a fiber in a single-layer protective coating; (**c**) is a fiber in a two-layer protective coating; 1 is the light-conducting core; 2 is the inner layer of the power rod; 3 is the outer layer of the power rod; and 4 is quartz glass.

A PMF is a heterogeneous structure consisting of doped fused silica. It is a special single-mode optical fiber that maintains the polarization of light. Panda optical fibers are related to PMFs (see Figure 1). A Panda fiber comprises not only differently doped fused silica elements, but also protective polymer coatings. The protective coating can be either single-layer or double-layer. The geometry of the coating depends on the materials and operating conditions.

The optical fiber preform is manufactured using Modified Chemical Vapor Deposition (MCVD) technology [29,30]. This makes it possible to introduce doping additives to the light-conducting core and power rods. The blanks of the light-conducting core and power rods are manufactured separately. These blanks undergo additional process steps to create the final fiber blank. The light-conducting core blank is built up with pure quartz layers to the required diameter. Holes for power rods are drilled in the obtained blank. Pure quartz layers in the blank are peeled off as much as possible by applying stress to the rods. This is necessary to form the required preform geometry with the established position of the elements. The preform is assembled by inserting rods into a blank with a light-conducting core. After that, the preform is subjected to high-temperature drawing. The optic preform is subjected to the drawing process at the fiber draw tower at high temperatures [31]. The macro-sized preform is drawn into a thin optical fiber of microscopic size under its own weight as a result of softening [30]. During the drawing process, a protective coating is applied to the optical fiber [32]. The coating is polymerized by ultraviolet radiation. The process is technologically and economically efficient [33].

The thermomechanical and thermophysical behavior of the primary (DeSolite 3471-1-152A) and secondary (DeSolite DS-2015) polymer protective coating materials from DSM Desotech Inc. (Elin, IL, USA) were considered in these studies [34,35]. The above-mentioned materials are qualified as acrylates. Experimental studies formed the basis for numerical analogues of the materials, formulated within the scope of the viscoelasticity theory. Models of materials are of practical interest and can be used for predictive analysis of technological processes of manufacturing and operation of optical fibers.

### 2.2. Materials of Polymer Coating

A protective coating is applied to the fiber during the drawing process and is cured by UV radiation. These polymers absorb light well in the UV range. This affects the formation of specimens of the required quality. It is problematic to produce a homogeneous massive specimen with a uniform polymerization degree. UV-induced degradation of the material can occur in the outer layers when exposed sufficiently to polymerize the inner layers. Therefore, in the current work, the procedure was developed for creating film specimens close in size to the thickness of optical fiber coatings.

A study of the complex modulus on specimens of polymer protective coatings (PCs), DeSolite 3471-1-152A and DeSolite DS-2015, used in a number of fiberoptic applications, was carried out within the scope of the present work. Preliminary studies have shown that relaxation transitions fall within the operational temperature range from −60 to +60 °C [36]. DeSolite 3471-1-152A is in a highly elastic relaxed state at room temperature, and at temperatures below −40 °C, it begins a smooth transition to a glassy state. DeSolite DS-2015 is in a glassy relaxed state at room temperature, and at temperatures above 30 °C, it begins a smooth transition to a highly elastic state. Therefore, viscoelasticity models must be used to correctly describe the thermomechanical response of such materials to thermal force effects. This is because thermoelastic models cannot adequately describe the materials’ behavior in the temperature ranges around relaxation transitions.

The viscoelastic behavior of the materials was studied on films with characteristic thicknesses comparable in values to those of a real coating, and on plates when the rigidity of the films was insufficient.

### 2.3. Experimental Study

An experimental study of the thermomechanical behavior of protective coating materials was carried out using the equipment of the Plastic Laboratory (Perm National Research Polytechnic University, Perm, Russia).

The study of the physical and mechanical properties of the polymer coatings DeSolite 3471-1-152A and DeSolite DS-2015 was carried out using a DMA Q800 dynamic mechanical analyzer (TA Instruments Inc., New Castle, DE, USA) with a GCA (gas cooling accessory) liquid nitrogen cooling system.

#### 2.3.1. Specimen Preparation

Specimens in two options were manufactured for this study: films up to 250 µm in thickness and plates with characteristic dimensions of 2 × 6 × 20 mm. The starting materials were photopolymers DeSolite DS-2015 and DeSolite 3471-1-152A (in a liquid, uncured state).

The specimens in the form of plates were produced in the following sequence: before casting into molds, the polymer was heated in a furnace to a temperature from +35 to +40 °C to reduce viscosity. Then, after casting into fluoroplastic molds, the specimens were polymerized by UV radiation with an exposure of at least 1 J/cm^2^. The obtained blanks were kept for several days at room temperature and then placed into a DMA Q800 thermal chamber and kept for 2 h at 80 °C (DeSolite 3471-1-152A) and 110 °C (DeSolite DS-2015) to relieve residual stress. After that, they were naturally cooled for several hours in a closed thermal chamber to room temperature. Specimens 6.25 mm wide were formed from the films using a cutting press.

Additionally, film specimens were produced using the method described below. The specimens were polymerized with a DRT-400 UV lamp (Open Joint-Stock Company LISMA—All-Russian Research, Design and Technological Institute of Light Sources named after A.N. Lodygin, Saransk, Russia). The working platform was positioned so as to ensure a radiation intensity on the specimen surface of at least 5 mW/cm^2^. The radiation intensity was measured using a TKA-01/3 luxometer–UV radiometer (LLC Scientific and Technical Enterprise TKA, St. Petersburg, Russia). The following preliminary operations were carried out in advance: 1–2 drops of the OP-7 auxiliary substance (nonylphenol ethoxylates-based surface-active agent) were applied to a substrate made of polyethylene terephthalate film and rubbed with a cotton cloth so that an extremely thin layer of the substance stayed on the surfaces of the films. A small amount of the composition was applied to the treated substrate and then covered with another layer of film and pressed down with a weight to distribute the liquid composition in a thin layer from 0.05 to 0.25 mm. After removing the load, the blank was placed in a radiation zone for the time required to form the exposure specified by the manufacturer; then, the polyethylene terephthalate film was removed from the cured polymer.

**Figure 2:**
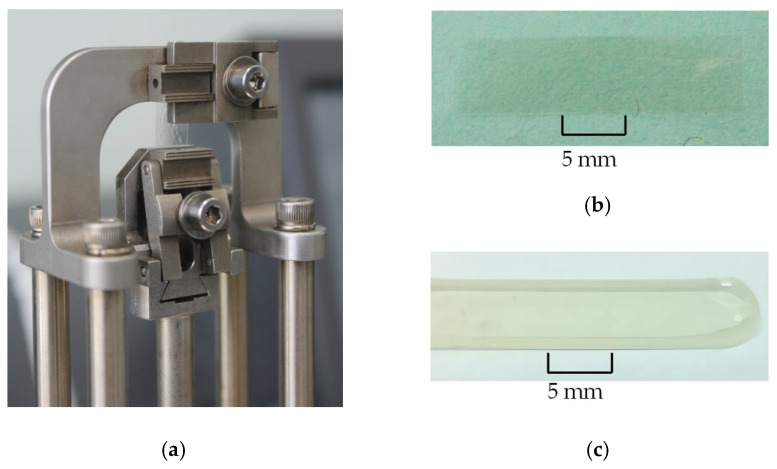
Experimental studies: (**a**) is the experimental equipment with accessories; (**b**) is the film specimens; and (**c**) is the plate specimens.

The obtained specimens, as well as the equipment used in the experiments, are presented in Figure 2. The typical dimensions of the samples were as follows: a length from 10 to 14 mm, a width from 6 to 6.25 mm, and a thickness from 0.05 to 2 mm.

This was due to the need to conduct experiments with different geometries of the specimens due to the sensitivity of materials to loading conditions, especially in temperature zones close to the structural and relaxation transitions of polymers.

Two types of samples were needed to conduct the experiments correctly: the films with characteristic dimensions of 10 × 6.25 × 0.05 mm (Figure 2b), and the plates with characteristic dimensions of 14 × 6 × 2 mm (Figure 2c).

#### 2.3.2. Experiment Setup

The studied polymers are in different relaxation states at room temperature. DeSolite 3471-1-152A is in a rubbery state. DeSolite DS-2015 is in a glassy state. According to the manufacturer’s data [34,35], relaxation transitions in protective coating polymers occur in the operating temperature range from −60 to +60 °C. However, the criterion that determines the relaxation transition area by storage modulus change from 1000 to 100 MPa provides only an approximate estimate. The width of the temperature range in which the relaxation state of the material changes is described approximately. To determine the range of temperature change required for a qualitative description of the thermomechanics of the materials, pilot experiments were carried out.

Transition of DeSolite 3471-1-152A from a rubbery to glassy state takes place at temperatures close to the lower limit of the range. According to the manufacturer’s data, glass transition occurs at temperatures from −54 °C to −65 °C [34]. Pilot experiments also demonstrated that the temperature range in which the glass transition occurred was less than −60 °C according to signals $E^{\prime }$ and $E^{\prime \prime }$ [36]. The studied temperature range was extended in the area of negative temperatures to −80 °C, and in some experiments, the specimens were cooled below −105 °C. This was performed to more fully describe the viscoelastic behavior.

DeSolite DS-2015 begins to devitrify in the upper part of the operating temperature range. Glass transition occurs at temperatures from +40 to +80 °C, as specified by the manufacturer [35]. According to the pilot experiments’ results [36], the range of the temperatures studied for DeSolite DS-2015 should be extended to +110 °C.

The temperature range selection for the thermomechanics studies of the protective polymers coating was carried out as part of a comprehensive assessment of signals $E^{\prime }$, $E^{\prime \prime }$, and tanδ based on the pilot experiments results.

In both cases, extending the temperature range studied is necessary to cover the entire relaxation transition range. This will allow for the accurate determination of the long-term and instantaneous elastic moduli and, consequently, the correct description of the viscoelastic behavior of the material.

To ensure the statistical significance of the results, at least 3 tests were conducted for each experiment.

### 2.4. Numerical Simulation and Its Realization

The numerical simulation was implemented on the basis of the Laboratory of Digital Engineering of the Advanced Engineering School “High School of Aircraft Engine Production” (Perm National Research Polytechnic University, Perm, Russia). Computing stations with an Intel Core i9-10900F processor, 128 GB of RAM, and 4 Tb of SSD were used for the numerical solution of the problem.

#### Thermomechanics of Panda-Type Optical Fiber

**Figure 3:**
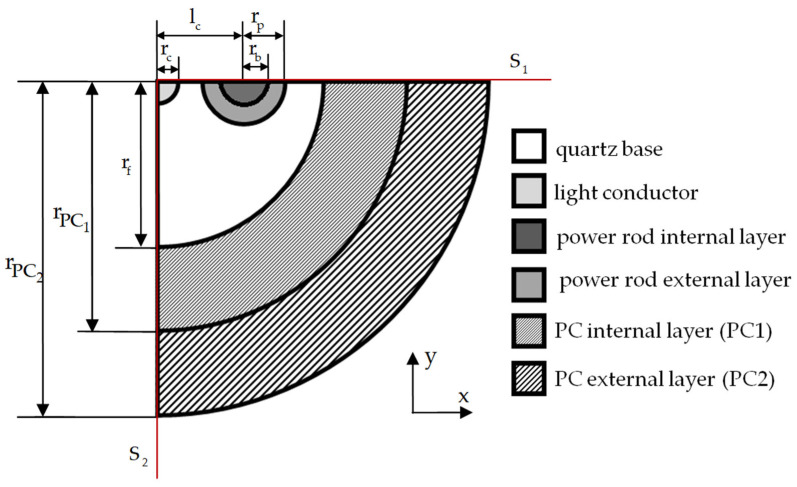
Design scheme of a fiber with a two-layer protective coating.

Figure 3 presents the design diagram of a Panda-type optical fiber in a two-layer protective polymer coating.

The Panda-type optical fiber model is as follows: the quartz base is SiO_2_; the light conductor is quartz-doped GeO_2_; the power rod internal layer is quartz-doped B_2_O_3_; the power rod external layer is quartz-doped B_2_O_3_ and P_2_O_5_; the PC internal layer is made of a polymer material (PC1 is DeSolite 3471-1-152A); and the PC external layer is made of a polymer material (PC2 is DeSolite DS-2015). Within the scope of the present work, the ideal geometric cross-section of a Panda-type optical fiber was simulated. The geometric parameters of the design diagram are presented in Table 1.

The thermoelastic properties of the materials were previously described in [36]. A viscoelastic model of the protective coating materials, taking into account the thermal expansion coefficient dependence on temperature, is considered in the current work. The simulation was implemented in the ANSYS Mechanical APDL 2021R2 software suite (Livermore, CA, USA).

**Figure 4:**
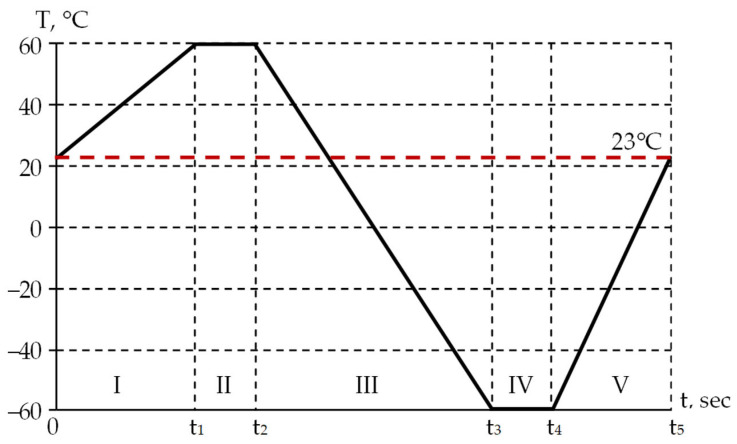
Temperature cycle. The values of the t_1_ … t_5_ parameters are presented in Table 2.

Heating and cooling of the optical fiber were performed according to the specified cycle (Figure 4). The thermal cycle was implemented in the operating temperature range of the optical fiber and included the following zones: I—heating from +23 to +60 °C, II—exposure for 30 s at the maximum positive temperature of +60 °C, III—cooling from +60 to −60 °C, IV—exposure for 30 s at the maximum negative temperature of −60 °C, and V—heating from −60 to +23 °C. Three thermal cycles with different heating and cooling rates were considered. The duration of each thermal cycle is presented in Table 2. The task was implemented to evaluate the operation of the viscoelastic behavior model of the protective coating materials.

The standard operating temperature range of fiber optic gyroscopes is from −60 to +60 °C, according to open-source data [37]. A range of FOG temperature changes was selected for the model performance analysis. A single thermal cycle with exposure at the maximum and minimum operating temperatures allows for the analysis of the fiber thermomechanics [6,38]. The rate of temperature change is the second parameter for evaluating the model performance. The decision to select three variants of changing the fiber heating/cooling rate for the first approximation analysis of the model performance was made within this study. Table 2 reflects the thermal cycle time intervals for different fiber heating/cooling rates.

Ideal contact is realized at the interface of the optical fiber elements $u_{n}^{1}{=u}_{n}^{2}$, $u_{\tau }^{1}{=u}_{\tau }^{2}$, $\sigma_{n}^{1}=\sigma_{n}^{2}$, and $\sigma_{n\tau }^{1}=\sigma_{n\tau }^{2}$, where 1 and 2 are symbols of the interface bodies, $u_{n}$ and $u_{\tau }$ are normal and tangential displacements, and $\sigma_{n}$ and $\sigma_{n\tau }$ are normal and tangential stresses.

Free thermal heating/cooling of the optical fiber in a planar configuration is considered. The fiber symmetry conditions are taken into account in the model. The boundaries with discarded parts of the system are fixed to implement the flat cross-sections hypothesis: $u_{y}=0$ at $\vec{x}\in S_{1}$; $u_{x}=0$ at $\vec{x}\in S_{2}$.

The convergence of the finite-element solution of the problem was formerly studied in the context of the problem of optical fiber contact with a polished metal surface [36]. The finite-element splitting of the free thermal heating/cooling model is performed similarly. The characteristic dimension of glassy elements is $r_{c}/10$. The characteristic dimension of the polymer coating elements is $r_{c}/30$.

## 3. Results and Discussion

### 3.1. Experimental Studies

#### 3.1.1. Glass Transition Temperature

**Figure 5:**
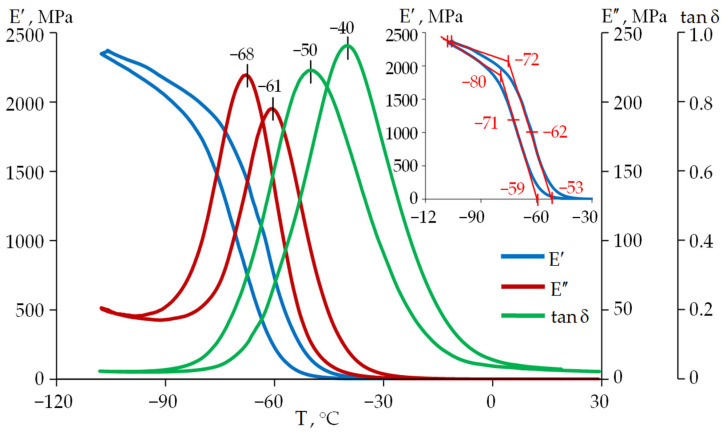
Temperature dependencies of the storage modulus $E^{\prime }$, loss modulus $E^{\prime \prime }$, and mechanical loss tangent tanδ for DeSolite 3471-1-152A at a frequency of 1 Hz and a cooling and heating rate of 2 °C/min.

**Figure 6:**
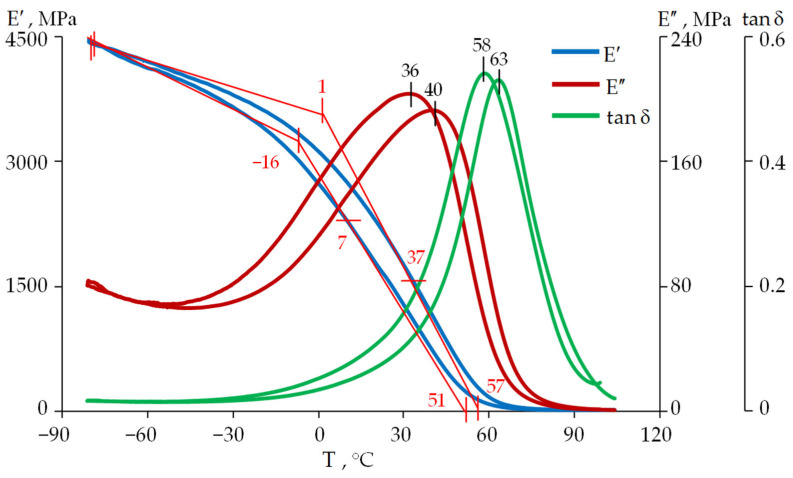
Temperature dependencies of the storage modulus $E^{\prime }$, loss modulus $E^{\prime \prime }$, and mechanical loss tangent tanδ for DeSolite DS-2015 at a frequency of 1 Hz and a cooling and heating rate of 2 °C/min.

The glass transition/devitrification process takes place in a temperature range and is accompanied by a sharp change in a number of physical properties of the material: heat capacity, thermal conductivity, viscosity, compressibility, etc. The relaxation transition can be characterized by a change in the deformation pattern of a specimen. The term “glass transition temperature” is a considerably formal term. It was introduced for the convenience of comparing materials. Its value is not a material constant, like the phase transition temperature (melting point, crystallization temperature, etc.), and depends on the kinematic characteristics of the loading. As an example, the curves $E^{\prime }$, $E^{\prime \prime }$, and tanδ for the two temperature change rates (+2 °C/min and −2 °C/min) are shown in Figure 5 and Figure 6. The curves shift to the right along the temperature axis during heating and to the left during cooling. The inflection point of the storage modulus curve $E^{\prime }$ in the relaxation transition region is considered to be the glass transition temperature in accordance with ISO 6721-11:2012 (“Plastics—Determination of dynamic mechanical properties—Part 11: Glass transition temperature”) [39,40]. The glass transition temperature is also determined by the peaks on the loss modulus curve $E^{\prime \prime }$ and the mechanical loss tangent tanδ [40,41]. The value at which the viscosity η = 10^12.3^ Pa·s is taken as the glass transition temperature (T_g_) when measuring the rheological properties of the materials [42,43]. The temperature at which the relaxation time is 100 s is assumed to be the same as in dielectric spectroscopy [44]. As a rule, different glass transition temperature values correspond to different evaluation criteria. Using several evaluation criteria makes it possible to estimate the width of the temperature range in which the relaxation transition occurs. It is also useful for interpreting the processes taking place in the polymer.

Determination of the glass transition temperatures and the relaxation transition width of the protective coating materials was performed by measuring dynamic mechanical properties using the method of linear scanning in heating mode in accordance with the ISO 6721 series of standards.

The experimental results for DeSolite DS-2015 and DeSolite 3471-1-152A are presented in Figure 5 and Figure 6, respectively. Glass transition temperatures determined by some of the above-mentioned criteria, for both heating and cooling modes, are shown in the diagrams.

The range of change in the glass transition temperatures of the optical fiber protective coating materials, determined by different criteria, is fairly wide. This is associated with the influence of many factors on the behavior of polymer materials and different approaches to determining the glass transition temperature [45,46].

#### 3.1.2. Viscoelastic Behavior of the Materials

**Figure 7:**
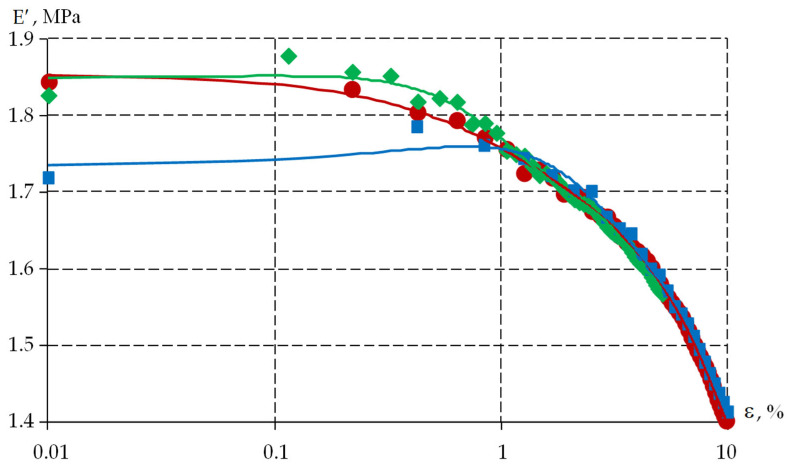
$E^{\prime }$ vs. strain amplitude dependence diagram for DeSolite 3471-1-152A at a frequency of 1 Hz and a temperature of T = 30 °C: red is specimen 1; green is specimen 2; and blue is specimen 3.

The viscoelastic response of polymers to deformation depends on temperature and loading history [47,48]. Natural experiments providing various deformation modes are necessary for the identification of properties [48,49]. Kinematic action on the polymer in accordance with the specified program is one of the options for such tests. The loading mode, in which the deformation of the specimen was set, varying in time according to the harmonic law $\varepsilon \left(t\right)=\varepsilon_{0}\sin\omega t$, where $\varepsilon_{0}$ is the amplitude, ω is the circular frequency (ω=2πν), and ν is the linear frequency, was implemented on the DMA Q800 as part of this study. A series of experiments was carried out in the uniaxial tension mode of specimens with a frequency in the range from 1 to 25 Hz at different temperatures. The $\varepsilon_{0}$ value was determined from a series of pilot experiments in order to estimate the limit of the tension deformations in the context of the linear viscoelasticity theory. The experiments were performed in accordance with the ISO 6721 series of standards. The specimens in the form of films and plates were subjected to stretching at a frequency of 1 Hz with increasing strain amplitude within the operating temperature range. The characteristic dependence of the storage modulus $E^{\prime }$ vs. strain amplitude is shown in Figure 7. The results are given for DeSolite 3471-1-152A at a frequency of ν=1 Hz and a temperature of T = 30 °C.

Analysis of the obtained dependencies demonstrated that the properties of the protective coating polymers were characterized by the linear viscoelasticity theory at tensile strains of up to 2%.

The dependence of the storage modulus $E^{\prime }$ vs. deformation can be described by a sixth-order polynomial. For deformations of 1 to 10%, the discrepancy between the parameter values for the three specimens considered is insignificant. Specimen number 2 withstood a significantly lower level of deformation. Specimen number 3 at the strain level of loss of 1% had significant differences from the other two considered specimens. The sensitivity of the material to deformation was previously identified in [50]. The material is sensitive to loading conditions.

Pilot experiments also demonstrated that increasing the load action frequency at a constant temperature resulted in an increase in the dynamic elastic modulus by approximately 1–2 orders of magnitude. A decrease in the temperature within the considered range resulted in a change in the modulus by about three orders of magnitude. The force generated by the device was insufficient in the subzero temperature range due to the rigidity of the DeSolite 3471-1-152A specimens in the form of plates. Quite the reverse, film specimens were too yieldable in high-temperature regions and did not provide acceptable measurement accuracy over the entire frequency range being considered.

Therefore, specimens of different thicknesses and geometries were used to collect data on the properties of the materials. Specimens in the form of plates with characteristic dimensions of 14 × 6 × 2 mm were studied in the temperature range from −20 to +80 °C. Film-shaped specimens with characteristic dimensions of 10 × 6.25 × 0.1 mm were studied in the temperature range from −80 to −20 °C.

DeSolite DS-2015 proved to be more resistant to temperature changes. Specimens in the form of films with characteristic dimensions of 10 × 6.25 × 0.05 mm were used for studying DeSolite DS-2015 over the entire frequency and temperature ranges.

**Figure 8:**
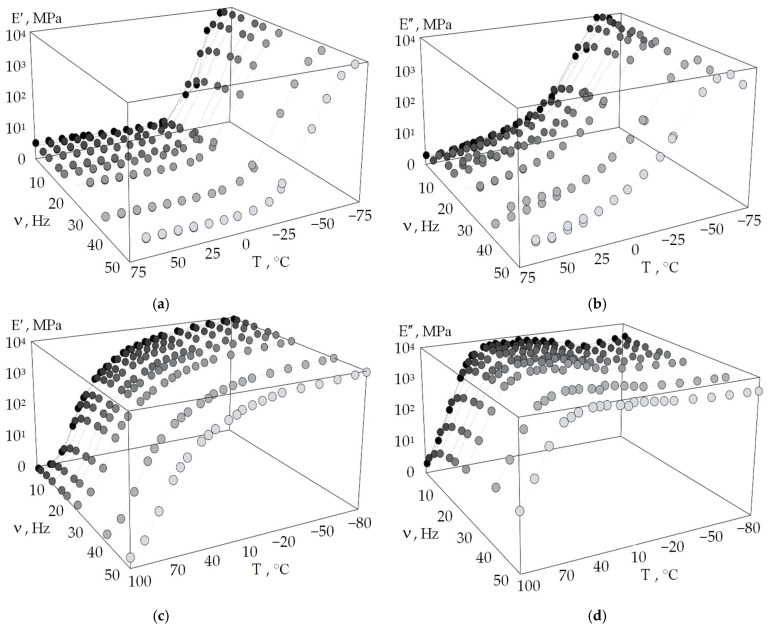
Experimental data of the dependence of the storage modulus (**a**,**c**) and loss modulus (**b**,**d**) on frequency and temperature: (**a**,**b**) are for DeSolite 3471-1-152A; (**c**,**d**) are for DeSolite DS-2015.

Discrete dependencies of the storage and loss moduli on temperature and frequency for DeSolite 3471-1-152A and DS-2015 were obtained as a result of a series of tests on DMA Q800 (Figure 8).

The obtained data of the dependence of the storage modulus and loss modulus on the temperature and frequency of the kinematic action correspond qualitatively to the known data for other cross-linked polymers [51]. This is also consistent with the data presented in Figure 6 and Figure 7. It can be noted that studies of the thermomechanical properties of polymers are often carried out in a wide temperature range. However, often, these are only negative [52] or positive [53,54] temperature ranges at constant frequencies. The dependencies of the storage modulus and loss modulus are in good agreement with the main functions of the viscoelastic materials [51].

It should be noted that the maximum values of the loss modulus of DeSolite 3471-1-152A are in the negative-temperature region. The glass transition of the material occurs at temperatures close to −60 °C, at which point the material loses its mobility, and its structure loses its ability to undergo large deformations. This can negatively affect the behavior of the optical fiber and signal transmission quality, since in such a state, the protective coating no longer protects the optical fiber from power actions. A similar maximum of DeSolite DS-2015 is in the positive temperature range of +20 to +60 °C, and with an increase in the frequency of exposure, it shifts to the left. These results allow us, for example, to assume that if such fibers are used as acoustic sensors, in the regions of maximum loss modulus, their sensitivity will decrease, which is due to the more efficient dissipation of oscillation energy in polymers due to dissipative effects.

The dependence of the storage modulus and loss modulus on the deformation frequency is nonlinear for protective coating materials. This is due to the rheology of the polymer and the structural state changes of the material. The storage and loss moduli reach a plateau with increasing frequency, which is typical for many viscoelastic materials [55,56].

The experimental data allowed us to determine the range of linear viscoelasticity (deformations of up to 2%), as well as to obtain information on the rheological characteristics and relaxation times spectrum. Based on the experimental studies, numerical analogues of the materials were formulated in the framework of relations of the generalized Maxwell model. Nonlinear behavioral models of the optical fiber protective coating materials are necessary to minimize the deviation of numerical simulation of the systems in which they are used from real objects [57].

#### 3.1.3. Thermal Expansion of the Materials

**Figure 9:**
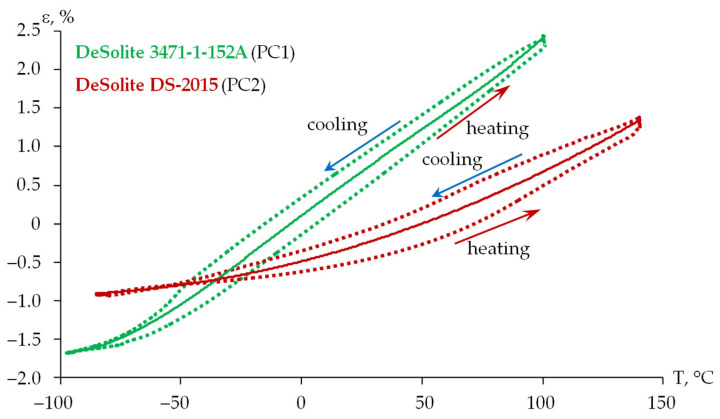
Dependence of the thermal deformation of the materials on temperature: dotted line is experimental data; solid line is polynomial approximation.

The thermal expansion coefficient (TEC) is considered a basic thermophysical characteristic reflecting the performance of the protective coating materials. The experiment was performed on film specimens at a constant tension force in the range from 0.001 to 0.005 N. This allowed us to maintain the straight geometry of the specimens, while introducing less than 5% error into the experimental data. The experimental studies and their verification are described in more detail in [58]. Deformation vs. temperature dependencies for protective coating materials were obtained after processing the experimental data (see Figure 9).

The approximating dependencies of thermal deformation on T are presented in the form of polynomials (1) for DeSolite 3471-1-152A and (2) for DeSolite DS-2015.

(1) $$\varepsilon_{\mathrm{PC}1}\left(T\right)=4.2387\times 10^{-9}\cdot T^{4}-3.097\times 10^{-7}\cdot T^{3}-1.6507\times 10^{-5}\cdot T^{2}+2.3536\times 10^{-2}\cdot T+0.1046$$

(2) $$\varepsilon_{\mathrm{PC}2}\left(T\right)=3.6391\times 10^{-5}\cdot T^{2}+7.9891\times 10^{-3}\cdot T-0.4892$$

The dependence of the TEC on temperature can be obtained by differentiating these dependencies.

### 3.2. Numerical Analogue of Polymer Protective Coating Materials

#### 3.2.1. Maxwell Model Based on Prony Series

For the mathematical description of the thermomechanical behavior of polymer products, taking into account the temperature change in a specified range and a strain history, the generalized Maxwell model was chosen [59]. The model uses an approximation of the relaxation function by an exponential Prony series [60,61,62].

The Maxwell model is suitable for describing highly viscous materials operating under long periods of loading. The model is well-suited to changes in material viscosity over time or under different temperature and force operating conditions [63]. Prony series are one of the common variants of constructing the Maxwell model [64]. The Maxwell viscoelastic model is widely used to describe stress relaxation and creep to estimate the viscoelastic properties of materials based on experimental data [65]. Accurate correspondence of material constants to physical phenomena is an important factor for describing materials [59]. The Maxwell model is effective because all its parameters have significant physical values related to the structure of a viscoelastic solid. The constitutive relation consists of a sufficiently large number of exponents that can describe the experimental data well enough. The model has a number of limitations [59,65]: increasing the exponent number can lead to mathematical models that have no physical meaning for the terms; the model is based on the assumption of a stepped load and an infinite loading rate. Despite its limitations, the model was chosen due to a number of advantages: it is simple enough to identify its material constants; it describes even complex relaxation processes well (except for rapid loading processes); it is implemented in the most popular CAE packages (ANSYS (ANSYS Inc., Livermore, CA, USA), ABAQUS (Dassault Systèmes Simulia Corp., Providence, RI, USA), and COMSOL (COMSOL Inc., Burlington, MA, USA), etc.); and it is suitable for constructing constitutive relations for a large number of materials.

The defining relations for the generalized Maxwell model in integral form, assuming that bulk relaxation in the materials is negligible, are of the following form [62]:

(3) $$\sigma =\int_{0}^{t}2G\left(t-\tau \right)\frac{\mathrm{de}}{d\tau }d\tau +B\Theta ,$$

wherein σ is the stress tensor; t and τ are the current and past time; $\hat{e}=\hat{\varepsilon }-\Theta \hat{E}/3$ is the strain deviator; $\hat{E}$ is the second-rank unit tensor; $\Theta =\varepsilon_{\mathrm{kk}}$ is the bulk deformation; and $G\left(t\right)$ is the shear relaxation function of the material, the approximation of which we express as a sum of exponentials:

(4) $$G=G_{\infty }+\sum_{i=1}^{n}G_{i}e^{-t/\tau_{i}^{G}},$$

(5) $$G=G_{0}\left(\alpha_{\infty }^{G}+\sum_{i=1}^{n}\alpha_{i}^{G}e^{-t/\tau_{i}^{G}}\right),$$

wherein i is the number of Prony series terms; $\alpha_{i}^{G}=G_{i}/G_{0}$ is the required approximation constants; $\tau_{i}$ is the relaxation time; and $G_{0}$ is the instantaneous shear modulus.

The long-term shear modulus $G_{\infty }$ and the corresponding coefficient $\alpha_{\infty }^{G}$ are obtained from the relation (5) at t=0:

(6) $$G_{0}=G_{0}\left(\alpha_{\infty }^{G}+\sum_{i=1}^{n}\alpha_{i}^{G}\right),$$

(7) $$\alpha_{\infty }^{G}=1-\sum_{i=1}^{n}\alpha_{i}^{G}.$$

The elastic (storage) and loss moduli in the context of the chosen model are of the following form:

(8) $$G^{\prime }=\alpha_{\infty }^{G}+\sum_{i=1}^{n}\left(\alpha_{i}^{G}{\left(\tau_{i}^{G}\omega \right)}^{2}\right)/\left(1+{\left(\tau_{i}^{G}\omega \right)}^{2}\right),$$

(9) $$G^{\prime \prime }=\sum_{i=1}^{n}\left(\alpha_{i}^{G}\tau_{i}^{G}\omega \right)/\left(1+{\left(\tau_{i}^{G}\omega \right)}^{2}\right).$$

To describe the temperature effect on the viscoelastic behavior of the materials, the Williams–Landel–Ferry equation [66] is used, with the help of which we introduce the relaxation time vs. temperature dependence into relations (8) and (9):

(10) $$\tau_{i}\left(T\right)=\tau_{i}\left(T_{r}\right)/A\left({T,T}_{r}\right),$$

(11) $$\log A\left({T,T}_{r}\right)=C_{1}\left(T-T_{r}\right)/\left(C_{2}+\left(T-T_{r}\right)\right)$$

wherein $C_{1}$ and $C_{2}$ are material constants; $T_{r}$ is the reference temperature.

Multicriteria optimization problems satisfying the condition of minimum discrepancy between experimental and calculated data were solved to find the constants $C_{1}$, $C_{2}$, and $\alpha_{i}^{G}$ by the Nelder–Mead method [67]. Thus, approximations of the discrete dependences were obtained.

A numerical procedure for solving the optimization problem using the Nelder–Mead method, varying the number of series terms from 2 to 100, was used. The procedure automatically performs the search. The number of series terms and model parameters is refined until the deviation between the simulated experimental conditions and the experimental data is less than 5%.

The optimal solution for the DeSolite 3471-1-152A material was found for i=18 (18 Prony series terms) at the selected reference temperature $T_{r}=-70$ °C. $C_{1}=20.036$, $C_{2}=32.666$, and $\alpha_{i}^{G}$ are presented in Table 3.

The optimal solution for the DeSolite 3471-1-DS-2015 material was found for i=60 (60 Prony series terms) at the selected reference temperature $T_{r}=0$ °C. $C_{1}=40,242.2827$, $C_{2}=267,448.888$, and $\alpha_{i}^{G}$ are presented in Table 4.

**Figure 10:**
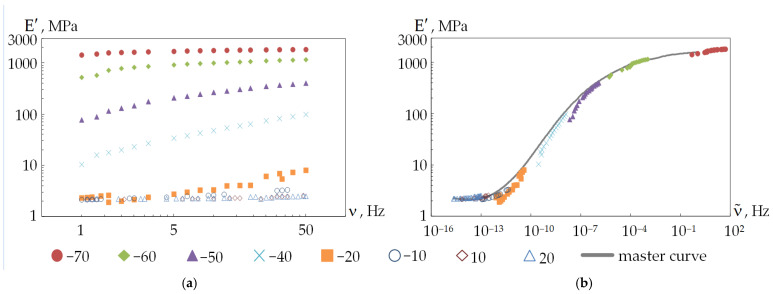
Processing the results based on the temperature–time analogy principle: (**a**) is the initial data; (**b**) plots a master curve based on experimental data at the equivalent oscillation frequency.

**Figure 11:**
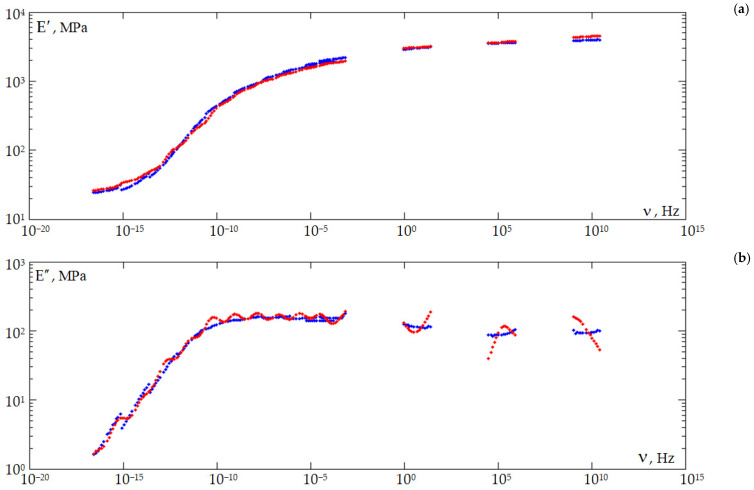
Dependence of the storage modulus (**a**) and loss modulus (**b**) on frequency (for DeSolite DS-2015): blue is experimental data; red is an approximation of the experimental data.

A master curve can be plotted based on the Williams–Landel–Ferry (WLF) temperature–time analogy principle using the obtained dependencies of $E^{\prime }$ and $E^{\prime \prime }$ on frequency [66,68]. The master curve describes the behavior of the material over a wide frequency (time) range. According to the WLF principle, there is a relationship between the frequency (time) and temperature dependence of the viscoelastic properties of the material. The procedure for processing the mechanical test results is illustrated in Figure 10. The result of processing the experimental data, taking into account the previously defined constants, is shown in Figure 11.

The nonlinear dependence of the storage modulus and loss modulus on the oscillation frequency during DMA, while reaching a plateau, is a characteristic feature of the cross-linked polymer rheology in the linear viscoelasticity region [56].

**Figure 12:**
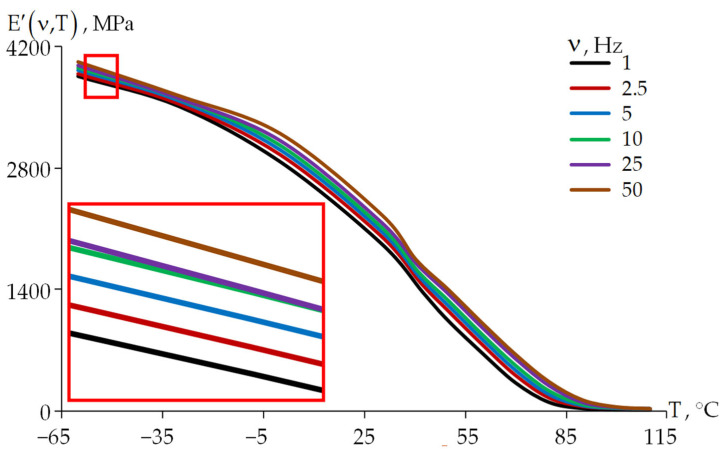
Dependence of E′ on temperature (for DeSolite DS-2015).

The dependency of relaxation transition on frequency for the DeSolite DS-2015 material is illustrated in Figure 12. It can be seen that with increasing frequency, the relaxation transition shifts to the right along the temperature scale.

The storage modulus of DeSolite DS-2015 at temperatures T ≥ +85 °C decreases by three orders of magnitude, approaching 2 MPa for all the considered oscillation frequencies of the specimens. The oscillation frequency has a greater influence on the storage modulus at high temperatures; so, at sub-zero temperatures, an increase in the oscillation frequency by 50 times leads to an increase in the storage modulus by no more than 5%. The oscillation frequency has the maximum effect on the storage modulus at temperatures above +60 °C; at temperatures from 80 to 90 °C, the storage modulus increases almost threefold, with an increase in the oscillation frequency from 1 to 50 Hz.

#### 3.2.2. Dependence of Thermal Deformation on Temperature

A user-defined program module was developed that allows setting the dependence of the coating material thermal deformation on temperature. The program makes it possible to recalculate an intercept depending on the choice of the initial stress-free state of the structure using Formulas (1) and (2).

**Figure 13:**
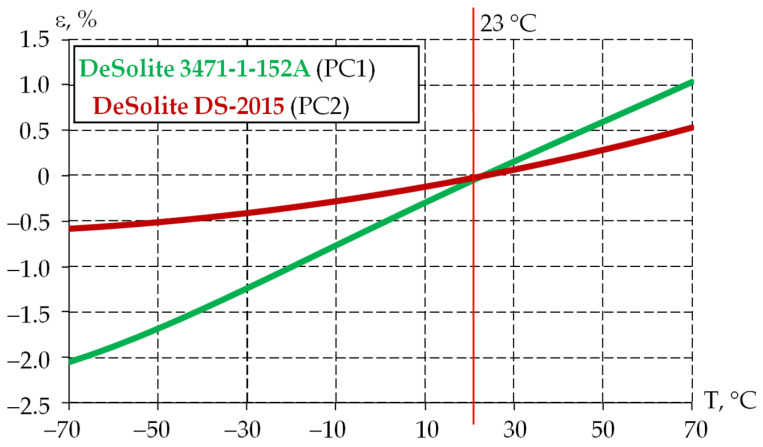
Dependence of thermal deformations on temperature (according to the numerical simulation data of the materials).

The dependencies of thermal deformation on temperature for polymer protective coating materials integrated into the viscoelastic model of the material are shown in Figure 13. The structure in the stress-free state is at 23 °C. For this purpose, intercept values in Formulas (1) and (2) are taken as −0.5307 and −0.203, respectively.

#### 3.2.3. Verification of the Numerical Analogue of Material Behavior

**Figure 14:**
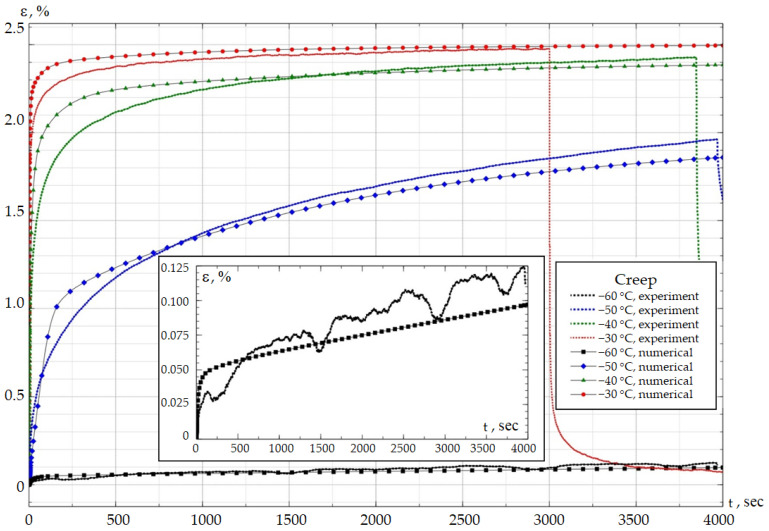
Deformation vs. time dependencies for DeSolite DS-2015 obtained during empirical and numerical studies.

A series of natural creep experiments at a constant load and different temperature values were carried out to verify the obtained defining relations (Figure 14). The data obtained were compared with the numerical calculation results; the comparison demonstrated a fairly good agreement. The results are presented for the external protective coating material DeSolite DS-2015. The range of negative operating temperatures was considered.

Temperature has a significant effect on the deformation level in the specimens. At a temperature of −60 °C, the deformation level does not exceed 0.1%; at −30 °C, it is 2.4%. The creep curve reaches an asymptote at a temperature of −30 °C, i.e., at times of more than 2000 s, all relaxation processes are completed. This effect is not observed at temperatures below −30 °C. The slowdown of relaxation processes with an increase in negative ambient temperatures is associated with glass transition processes. The dependencies of creep deformation on time, as well as the characteristic phenomena, are consistent with studies of polymer materials of various types [69,70,71].

No restrictions were imposed on the term number in the Prony series. This resulted in a significant difference in the term numbers for the materials under consideration. The numerical identification procedure is based on the inverse problem. This suggests that the solution is not unique, but rather one of several possible ones. Creep experiments confirm that the current model adequately describes the thermomechanics of DeSolite DS-2015. The material models will be refined as a development of this study using an improved numerical identification procedure [72]. An analysis of the feasibility of describing the thermomechanics of materials using the special case, the classical, and the modified Anand models is also planned as a further development of this study [73].

### 3.3. Numerical Model of Thermomechanics of Panda-Type Optical Fiber Protective Coatings

As part of the current work, the application of the developed viscoelastic models of the protective coating materials was considered. The thermal expansion coefficient of the protective coatings was considered in two ways: as a constant coefficient, $200\cdot 10^{-6}$ K^−1^ and $50\cdot 10^{-6}$ K^−1^ for PC1 and PC2, respectively [36], and as a temperature-dependent coefficient, characterized by the plot in Figure 13.

**Figure 15:**
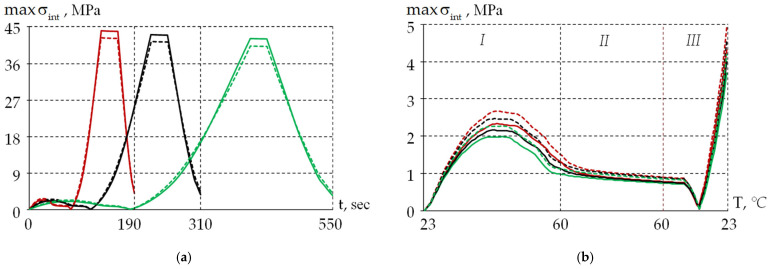
Dependencies of the maximum stress intensity in the external protective coating: (**a**) is the dependence on time; (**b**) is the dependence on temperature. Red line is cycle 1; black line is cycle 2; green line is cycle 3; dashed line is constant TEC; and solid line is temperature-variable TEC.

The dependencies of the stress–strain state on time and temperature were obtained for each thermal cycle shown in Table 2. The dependencies of the stress intensity maximum value in the external polymer coating (PC2) on time and temperature are presented in Figure 15 The faster the heating/cooling processes occur, the higher the stress level in the material (see Figure 15a). The maximum stress level is achieved at subzero temperatures in zone III. The decrease in the maximum stress level from cycle 1 to cycle 3 does not exceed 2.25%. Visible differences in the material behavior with constant and variable TECs are observed only in zone III, by 3.77, 4.02, and 4.27% for cycles 1, 2, and 3, respectively. Stress relaxation in PC2 at −60 °C practically does not occur; the level comprises no more than 0.43%.

Figure 15b shows the dependence of stresses on temperature in the range not lower than +23 °C (zones I and II, and the starting area of zone III). The stress level here does not exceed 5 MPa. When heated (in zone I), the stress intensity increases up to a temperature of about +45 °C. When cooled (in zone III), the stress intensity increase begins at a temperature of approximately +50 °C.

**Figure 16:**
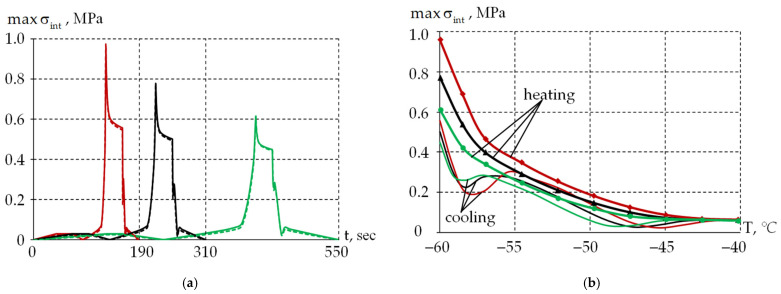
Dependencies of the maximum stress intensity in the inner protective coating: (**a**) is the dependence on time; (**b**) is the dependence on temperature; red line is cycle 1; black line is cycle 2; and green line is cycle 3. Dashed line is constant TEC; solid line is temperature-variable TEC.

The dependencies of the stress intensity maximum value in the inner polymer coating (PC1) on time and temperature are presented in Figure 16.

The faster the heating/cooling processes occur, the higher the stress level in the material (see Figure 16a). Taking into account that the TEC practically does not affect the stress–strain state behavior. The maximum stress level is achieved at a temperature of −60 °C, but only in the starting area of zone III, similar to PC2. Significant stress relaxation occurs during exposure at −60 °C. Table 5 shows the values of stress level reduction in zone III. The lower the heating/cooling rate, the smaller the stress reduction value during exposure at −60 °C.

The heating and cooling zones in the temperature range from −40 to −60 °C are shown in Figure 16b. Heating and cooling proceed differently. It is obvious that the optical properties of the system in certain cases will depend not just on the current temperature, but on the temperature change history.

## 4. Conclusions

This study developed a methodology for constructing a viscoelastic model using Maxwell-type equations based on the Prony series for polymeric optical fiber protective coating materials. The model is based on experimental DMA data in accordance with the ISO 6721 series of standards. DeSolite 3471-1-152A and DeSolite DS-2015, widely used as protective coatings for Panda-type optical fibers, were studied under different experimental conditions. Experiments were conducted both to develop and to validate the model. It is confirmed that the viscoelastic model based on the Prony series allows one to describe the thermomechanics of materials.

Defining relations for the considered polymers were formulated using the Maxwell model through multicriteria optimization by the Nelder–Mead method. The optimal solution was found for 18 series terms at the selected reference temperature $T_{r}=-70$ °C, $C_{1}=20.036$, and $C_{2}=32.666$ for the DeSolite 3471-1-152A material. The optimal solution was found for 60 series terms at the selected reference temperature $T_{r}=0$ °C, $C_{1}=40,242.2827$, and $C_{2}=267,448.888$ for the DeSolite DS-2015 material.

An extended temperature range within the experiments is required to detect the glass transition temperatures and relaxation transitions of materials: from −105 to 30 °C for DeSolite 3471-1-152A, and from −80 to 110 °C for DeSolite DS-2015.

The glass transition temperatures established according to a number of theories and the wide range of relaxation transitions confirm the complex materials’ thermomechanics.

The application of the obtained polymer behavior models was tested within the framework of free thermal heating/cooling of Panda-type optical fibers. This study was conducted for the fiber optical gyroscope operating temperature range of −60 to +60 °C. Three fiber heating/cooling rates were considered. The stress level depends on the temperature change rate: the faster the heating/cooling processes occur, the higher the stress level. It has been established that coatings have a significant impact on the optical fiber behavior, especially in the region of negative operating temperatures. The highest stress level in both polymers was observed at −60 °C.

Information on the thermomechanical properties of the materials makes it possible to determine the operating temperature range and to make a reasonable decision on the applicability of a particular structure, taking into account the operational characteristics. An accurate and reliable specification of the complex nonlinear behavior of polymer coatings as a function of temperature and strain rates is required to improve the accuracy of the interpretation of data obtained from the fiber optic sensors. Formulation of defining relations adequately predicting the thermomechanical behavior of the materials over a wide temperature range, including relaxation transitions, is urgent and necessary for the development of the scientific field. This work is of high practical importance in terms of accumulating data on the mechanical behavior of the coatings, including over a wide range of temperatures and force loads. The current study results also form a database for creating digital shadows and twins of optical fibers and monitoring systems in which they are used.

As a development of this work, a multifactorial analysis of the behavior of fibers in single-layer and two-layer protective polymer coatings under different thermal and force loads is currently being implemented.

## Figures and Tables

**Table 1:** Geometric parameters.

Parameter	Value	Parameter	Value
$r_{f}$	40 μm	$l_{c}$	15 μm
$r_{{\mathrm{PC}}_{1}}$	65 μm	$r_{p}$	7.5 μm
$r_{{\mathrm{PC}}_{2}}$	83.5 μm	$r_{b}$	3.5 μm
$r_{c}$	3 μm	$h_{\mathrm{bf}}{=r}_{p}{-r}_{b}$	4 μm

**Table 2:** Parameters of temperature cycles.

Temperature Cycle	t_1_, Sec	t_2_, Sec	t_3_, Sec	t_4_, Sec	t_5_, Sec
**Cycle 1**	40	70	130	160	190
**Cycle 2**	70	100	220	250	310
**Cycle 3**	130	160	400	430	550

**Table 3:** Material constants of the generalized Maxwell model for DeSolite 3471-1-152A.

i	$\alpha_{i}^{G}$	$\tau_{i}$	i	$\alpha_{i}^{G}$	$\tau_{i}$	i	$\alpha_{i}^{G}$	$\tau_{i}$
1	3.14 × 10^−3^	1.29 × 10^−4^	7	2.80 × 10^−1^	5.99 × 10^2^	13	4.43 × 10^−4^	2.78 × 10^9^
2	2.83 × 10^−2^	1.67 × 10^−3^	8	3.23 × 10^−2^	7.74 × 10^3^	14	1.06 × 10^−3^	3.59 × 10^10^
3	1.57 × 10^−1^	2.15 × 10^−2^	9	3.24 × 10^−1^	1.00 × 10^5^	15	2.07 × 10^−4^	4.64 × 10^11^
4	6.38 × 10^−4^	2.78 × 10^−1^	10	2.81 × 10^−2^	1.29 × 10^6^	16	8.22 × 10^−5^	5.99 × 10^12^
5	3.03 × 10^−2^	3.59 × 10^0^	11	6.21 × 10^−3^	1.67 × 10^7^	17	3.84 × 10^−5^	7.74 × 10^13^
6	6.28 × 10^−2^	4.64 × 10^1^	12	4.36 × 10^−2^	2.15 × 10^8^	18	2.30 × 10^−5^	1.00 × 10^15^

**Table 4:** Material constants of the generalized Maxwell model for DeSolite DS-2015.

i	$\alpha_{i}^{G}$	$\tau_{i}$	i	$\alpha_{i}^{G}$	$\tau_{i}$	i	$\alpha_{i}^{G}$	$\tau_{i}$	i	$\alpha_{i}^{G}$	$\tau_{i}$
1	4.86 × 10^−3^	1.03 × 10^−23^	16	3.35 × 10^−2^	6.99 × 10^−21^	31	2.08 × 10^−4^	4.76 × 10^−13^	46	3.38 × 10^−3^	3.24 × 10^0^
2	1.29 × 10^−2^	1.11 × 10^−23^	17	2.98 × 10^−2^	1.63 × 10^−20^	32	4.45 × 10^−3^	2.39 × 10^−12^	47	1.04 × 10^−1^	3.50 × 10^1^
3	3.59 × 10^−3^	1.26 × 10^−23^	18	3.73 × 10^−3^	3.98 × 10^−20^	33	7.41 × 10^−3^	1.26 × 10^−11^	48	1.49 × 10^−2^	3.98 × 10^2^
4	2.10 × 10^−4^	1.51 × 10^−23^	19	1.01 × 10^−2^	1.03 × 10^−19^	34	2.21 × 10^−2^	6.99 × 10^−11^	49	3.46 × 10^−2^	4.76 × 10^3^
5	4.25 × 10^−2^	1.90 × 10^−23^	20	2.34 × 10^−2^	2.78 × 10^−19^	35	3.33 × 10^−2^	4.08 × 10^−10^	50	3.49 × 10^−2^	5.99 × 10^4^
6	2.73 × 10^−3^	2.51 × 10^−23^	21	6.15 × 10^−3^	7.94 × 10^−19^	36	2.87 × 10^−3^	2.51 × 10^−9^	51	3.28 × 10^−2^	7.94 × 10^5^
7	5.56 × 10^−3^	3.50 × 10^−23^	22	9.26 × 10^−3^	2.39 × 10^−18^	37	2.04 × 10^−2^	1.63 × 10^−8^	52	3.62 × 10^−2^	1.11 × 10^7^
8	2.64 × 10^−2^	5.14 × 10^−23^	23	2.94 × 10^−2^	7.55 × 10^−18^	38	7.08 × 10^−3^	1.11 × 10^−7^	53	3.50 × 10^−2^	1.63 × 10^8^
9	5.99 × 10^−5^	7.94 × 10^−23^	24	5.20 × 10^−3^	2.51 × 10^−17^	39	2.68 × 10^−2^	7.94 × 10^−7^	54	3.37 × 10^−2^	2.51 × 10^9^
10	7.64 × 10^−3^	1.29 × 10^−22^	25	2.28 × 10^−3^	8.80 × 10^−17^	40	6.81 × 10^−4^	5.99 × 10^−6^	55	1.42 × 10^−2^	4.08 × 10^10^
11	1.74 × 10^−2^	2.21 × 10^−22^	26	4.68 × 10^−2^	3.24 × 10^−16^	41	1.31 × 10^−3^	4.76 × 10^−5^	56	7.62 × 10^−3^	6.99 × 10^11^
12	5.14 × 10^−3^	3.98 × 10^−22^	27	3.96 × 10^−2^	1.26 × 10^−16^	42	6.85 × 10^−3^	3.98 × 10^−4^	57	1.86 × 10^−3^	1.26 × 10^13^
13	8.18 × 10^−3^	7.55 × 10^−22^	28	2.22 × 10^−2^	5.14 × 10^−15^	43	5.09 × 10^−2^	3.50 × 10^−3^	58	1.11 × 10^−3^	2.39 × 10^14^
14	1.24 × 10^−2^	1.51 × 10^−21^	29	2.10 × 10^−3^	2.21 × 10^−14^	44	6.82 × 10^−3^	3.24 × 10^−2^	59	3.26 × 10^−4^	4.76 × 10^15^
15	3.28 × 10^−3^	3.16 × 10^−21^	30	1.39 × 10^−3^	1.00 × 10^−13^	45	3.36 × 10^−2^	3.16 × 10^−1^	60	5.48 × 10^−5^	1.00 × 10^17^

**Table 5:** Stress intensity reduction values in zone III.

TEC	Cycle 1	Cycle 2	Cycle 3
$\alpha_{\mathrm{constant}}$	76.83%	57.35%	38.28%
$\alpha \left(t\right)$	72.49%	54.26%	36.31%

## Data Availability

The original contributions presented in this study are included in this article. Further inquiries can be directed to the corresponding author.
